# 917. Development and Validation of an Automated Natural Language Processing Method for Identifying Postherpetic Neuralgia from Electronic Health Records

**DOI:** 10.1093/ofid/ofad500.962

**Published:** 2023-11-27

**Authors:** Chengyi Zheng, Bradley Ackerson, Leticia I Vega Daily, Jeannie Song, Lina S Sy, Lei Qian, Yi Luo, Ana Florea, Jennifer H Ku, Yanjun Cheng, Jun Wu, Hung Fu Tseng

**Affiliations:** Kaiser Permanente Southern California, Pasadena, California; Kaiser Permanente Southern California, Pasadena, California; Kaiser Permanente, Northridge, California; Kaiser Permanente Southern California, Pasadena, California; Kaiser Permanente Southern California, Pasadena, California; Kaiser Permanente Southern California, Pasadena, California; Kaiser Permanente Southern California, Pasadena, California; Kaiser Permanente Southern California, Pasadena, California; Kaiser Permanente Southern California, Pasadena, California; Kaiser Permanente South California, San Gabriel, California; Kaiser Permanente Southern California, Pasadena, California; Kaiser Permanente Southern California, Pasadena, California

## Abstract

**Background:**

Postherpetic neuralgia (PHN) is a debilitating complication of herpes zoster (HZ). PHN diagnosis codes tend to be inaccurate or incomplete. The criterion standard for identifying PHN in electronic health records (EHR) is manual chart review, which can be costly and time-consuming. We developed and validated a method to automatically identify PHN from free-text EHR data using natural language processing (NLP).

**Methods:**

This study utilized EHR data of patients aged ≥50 years who had an incident HZ diagnosis and associated antiviral prescription between 2018-2022 at Kaiser Permanente Southern California. Among patients with ≥1 encounter during the 90-180 days after the incident HZ diagnosis, PHN was defined as pain/discomfort consistent with the HZ episode between 90-180 days after the initial HZ diagnosis; the symptoms were at the location of the initial HZ rash and were not due to other obvious causes. Trained research associates and an infectious disease physician manually reviewed and identified PHN cases from EHR. From these reviewed HZ cases, we randomly selected 500 and 800 cases for NLP development and validation, respectively. We developed and validated an NLP algorithm to identify PHN based on our case definition. Using chart-reviewed results as the criterion standard, the accuracy of NLP-based results was compared with that of diagnosis code-based results (ICD-10 codes B02.22, B02.23, B02.29 from all clinical encounters during the 90-180-day period).

**Results:**

Compared to the criterion standard, the NLP algorithm achieved 100% sensitivity (code-based 64.8%), 99.9% specificity (code-based 97.0%), 98.6% positive predictive value (code-based 67.7%), and 100% negative predictive value (code-based 96.6%) in identifying PHN cases. In the validation data, the prevalence of PHN was 8.9% based on manual chart review. Although the prevalence of PHN identified by the two methods was similar (NLP-based 9.0%, code-based 8.5%), the code-based method misclassified more than one-third of chart-confirmed PHN cases.

Accuracy measurements of NLP and diagnosis codes in identifying PHN, as compared with chart-confirmed validation data.

**Figure:**
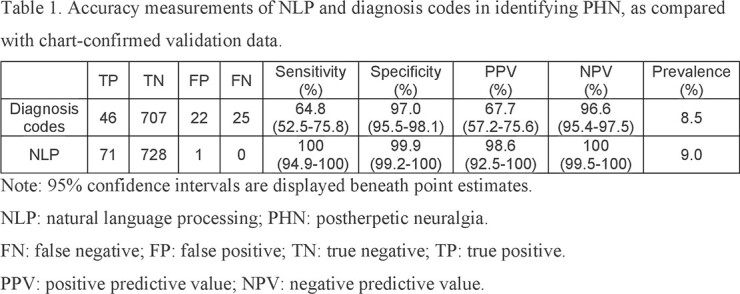

**Conclusion:**

We developed and validated an automated method to identify PHN cases using the clinical text from EHR of HZ patients with high accuracy. This method can be a valuable tool to facilitate population-based studies of PHN.

**Disclosures:**

**Bradley Ackerson, MD**, Dynavax: Grant/Research Support|Genentech: Grant/Research Support|GlaxoSmithKline: Grant/Research Support|Moderna: Grant/Research Support|Pfizer: Grant/Research Support **Leticia I. Vega Daily, MSW**, GlaxoSmithKline: Grant/Research Support **Jeannie Song, MPH**, GlaxoSmithKline: Grant/Research Support|Moderna: Grant/Research Support|Pfizer: Grant/Research Support **Lina S. Sy, MPH**, Dynavax: Grant/Research Support|GlaxoSmithKline: Grant/Research Support|Moderna: Grant/Research Support **Lei Qian, PhD**, Dynavax: Grant/Research Support|GlaxoSmithKline: Grant/Research Support|Moderna: Grant/Research Support **Yi Luo, PhD**, GlaxoSmithKline: Grant/Research Support|Moderna: Grant/Research Support|Pfizer: Grant/Research Support **Ana Florea, PhD MPH**, Gilead: Grant/Research Support|GlaxoSmithKline: Grant/Research Support|Moderna: Grant/Research Support|Pfizer: Grant/Research Support **Jennifer H. Ku, PhD MPH**, GlaxoSmithKline: Grant/Research Support|Moderna: Grant/Research Support **Hung Fu Tseng, PhD MPH**, GSK: Grant/Research Support|Moderna: Grant/Research Support

